# Positive resection margins in Crohn’s disease are a relevant risk factor for postoperative disease recurrence

**DOI:** 10.1038/s41598-024-61697-w

**Published:** 2024-05-11

**Authors:** Matthias Kelm, Clara Benatzky, Viktoria Buck, Anna Widder, Katrin Schoettker, Mathias Rosenfeldt, Markus Brand, Nicolas Schlegel, Christoph-Thomas Germer, Alexander Meining, Asma Nusrat, Sven Flemming

**Affiliations:** 1 Department for General, Visceral, Transplant, Vascular and Pediatric Surgery, University Hospital of Würzburg, Oberduerrbacher Str., 697080 Würzburg, Germany; 2 Department of Internal Medicine, Division of Gastroenterology, University Hospital of Würzburg, Würzburg, Germany; 3 Department of Pathology, University of Würzburg, Würzburg, Germany; 4 Department of Pathology, University of Michigan Medical School, Ann Arbor, USA

**Keywords:** Crohn’s disease, Postoperative disease recurrence, Ileocecal resection, Inflammatory bowel disease, Surgery

## Abstract

Postoperative disease recurrence in Crohn’s disease represents a relevant issue despite recent advancements in surgical and medical therapies. Additional criteria are necessary to improve the identification of patients at risk and to enable selective therapeutic approaches. The role of resection margins on disease recurrence remains unclear and general recommendations are lacking. A single-center retrospective analysis was performed including all patients who received ileocecal resection due to Crohn’s disease. Resection margins were analyzed by two independent pathologists and defined by histopathological criteria based on previous consensus reports. 158 patients were included for analysis with a median follow up of 35 months. While postoperative morbidity was not affected, positive resection margins resulted in significantly increased rates of severe endoscopic recurrence at 6 months (2.0% versus 15.6%, p = 0.02) and overall (4.2% versus 19.6%, p = 0.001), which resulted in significantly increased numbers of surgical recurrence (0% versus 4.5%, p = 0.04). Additionally, positive margins were identified as independent risk factor for severe endoscopic disease recurrence in a multivariate analysis. Based on that, positive margins represent an independent risk factor for postoperative endoscopic and surgical disease recurrence. Prospective studies are required to determine whether extended resection or postoperative medical prophylaxis is beneficial for patients with positive resection margins.

## Introduction

Crohn’s disease (CD) represents a challenging socio–economic burden for health care systems and patients worldwide. While the disease can be highly heterogenous regarding its clinical presentation, therapeutic strategies are usually multidisciplinary and include medical as well as surgical options. Importantly, despite medical advancements such as biologicals, rates of patients who suffer from (complicated) CD and require surgery remain relevant and stable over decades^1,2^. In case of isolated terminal ileitis, current guidelines recommend surgical resection as alternative to medical treatment for primary treatment^3^. This recommendation is mainly based on data from the LR!C-trial which were confirmed by novel studies^4–6^. Based on that, surgery represents not only a therapeutic option for complicated CD but also a reasonable alternative in case of localized terminal ileitis.

Despite the advantages of surgical resection compared to medical treatment in closely defined situations, postoperative disease recurrence at the site of the anastomosis represents an important issue following surgical resection^7^. Studies demonstrated relevant rates of disease recurrence with many patients needing additive immunosuppressive medication or re-operation over time^8–10^. To overcome this concern, various strategies have been evaluated to decrease rates of local disease recurrence. While one aspect represents the creation of the anastomosis, neither different orientations nor technical aspects showed advantages for one or the other strategy so far^11,12^. In contrast, novel concepts such as the Kono-S anastomosis or extended resection of the mesenterium demonstrate promising results to potentially decrease rates of the disease recurrence^11,13,14^, however, those strategies still need to be confirmed in ongoing prospective randomized trials.

Besides surgical strategies, another therapeutic aspect for disease recurrence represents to be pharmacological prophylaxis. However, current concepts of international guidelines on postoperative medical treatment are heterogenous and clear definitions of risk factors lacking^15,16^. To improve the identification of patients at increased risk for postoperative disease recurrence to enable selective medical prophylaxis, histopathological factors such as remaining inflammation at resection margins could be of relevant interest. While recent evidence is inconclusive with various studies demonstrating different outcomes^17–19^, no clear recommendation on the role of resection margins can currently be made. Therefore, the present retrospective analysis was conducted to assess the potential impact of positive resection margins for postoperative disease recurrence in a large patient cohort of patients suffering from localized CD.

## Materials and methods

### Study design

A retrospective single-center analysis of all patients who received ileocecal resection (ICR) due to CD at the department of general, visceral, transplant, vascular and pediatric surgery at the University Hospital of Wuerzburg, Germany between 2014 and 2021 was carried out. All patients suffering from terminal inflammatory (Montreal classification L1 and L3), penetrating and/or stricturing ileitis (Montreal classification B1–B3) were included. Operations were performed by three senior surgeons specialized in colorectal surgery.

### Data acquisition and study population

Patient baseline characteristics including age, sex, symptoms, previous medical and surgical history as well as sociodemographic patient characteristics were retrieved from the local database. Preoperatively, the extent of inflammation was assessed by endoscopy and MRI scan followed by an individual case discussion by a multidisciplinary IBD team including a gastroenterologist, surgeon, pathologist, and radiologist about the indication of surgery. To evaluate the role of the resection margins for disease recurrence, patients were divided into two groups according to the status of the resection margins (positive or negative for inflammation).

### Histopathological assessment of the resection margins

**Figure 1:**
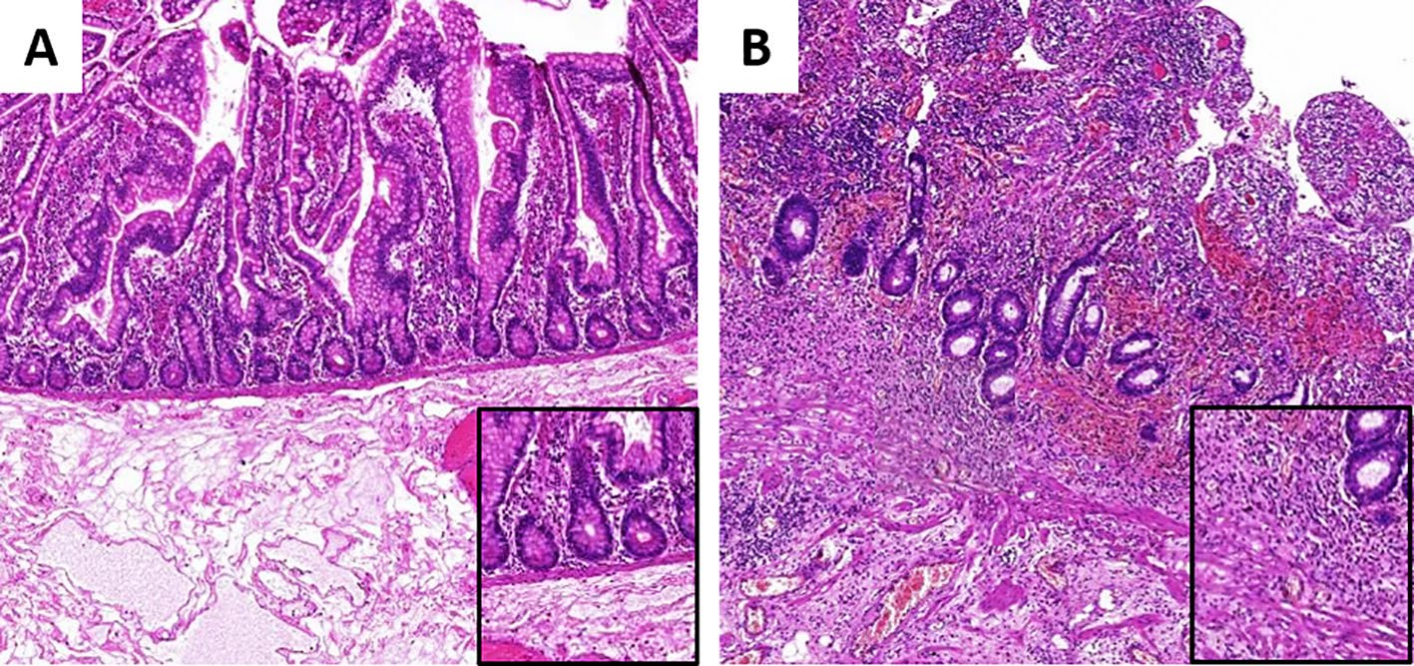
Histopathological images. (**A**) Negative resection margins. (**B**) Positive resection margins.

Surgical resection was performed in macroscopically non-inflamed areas. The microscopic status of the resection margin was evaluated by two independent pathologists. Histopathological criteria for microscopic disease were based on an international consensus adjusted from previous studies^20–22^. Selected criteria for positive resection margins were active inflammation characterized by neutrophils localized in the epithelium referred to as cryptitis, and/or plexitis (Fig. 1).

### Outcome

The primary outcome was the long-term rate of severe endoscopic disease recurrence (defined as Rutgeerts-score i3 and i4). The definition of severe endoscopic recurrence was based on previous studies^23,24^ and international guidelines^25^. Secondary outcomes were short-term endoscopic recurrence after 6 months as well as clinical and surgical recurrence including the need for postoperative immunosuppressive medication. The assessment of the Rutgeerts-scores was reviewed and scored for all patients by a senior gastroenterologist specialized in IBD. Surgical recurrence was defined as re-operation due to disease recurrence at the site of the anastomosis. In addition, short-term postoperative follow-up such as rates of surgical and non-surgical complications were also evaluated as secondary endpoints. For patients with positive resection margins, subgroup analyses were performed in regard of the role of the localization of positive resection margins (oral, aboral, both) as well as the effect of postoperative medical therapy on disease recurrence.

### Statistics

Descriptive data were evaluated and presented as median with range or total numbers with percentages. In univariate analyses, differences in patient characteristics as well as primary and secondary endpoints were assessed by *t*-test, Fisher’s exact test or ANOVA test in accordance with data scale and distribution. To identify independent risk factors for severe endoscopic recurrence, the Cox multivariate model was performed for a multivariate analysis and data were presented as hazard ratios (HR) with 95% confidence intervals (95% CI). For all analyses, a p-value of < 0.05 was considered statistically significant. Statistical analyses were performed using GraphPad Prism (version 8, Graph Pad Software, Inc., San Diego, USA) for descriptive analysis and SPSS statistics (version 28, IBM, Armonk, USA) for multivariate analysis.

### Ethical Approval

Ethical approval for the study was obtained from the Ethics Committee of the University of Wuerzburg, Germany (approval number: 2022070601). Informed consent was waived by Ethics Committee of the University of Wuerzburg, Germany. All analysis were done in accordance with relevant guidelines and regulations.

## Results

### Patient characteristics

**Figure 2:**
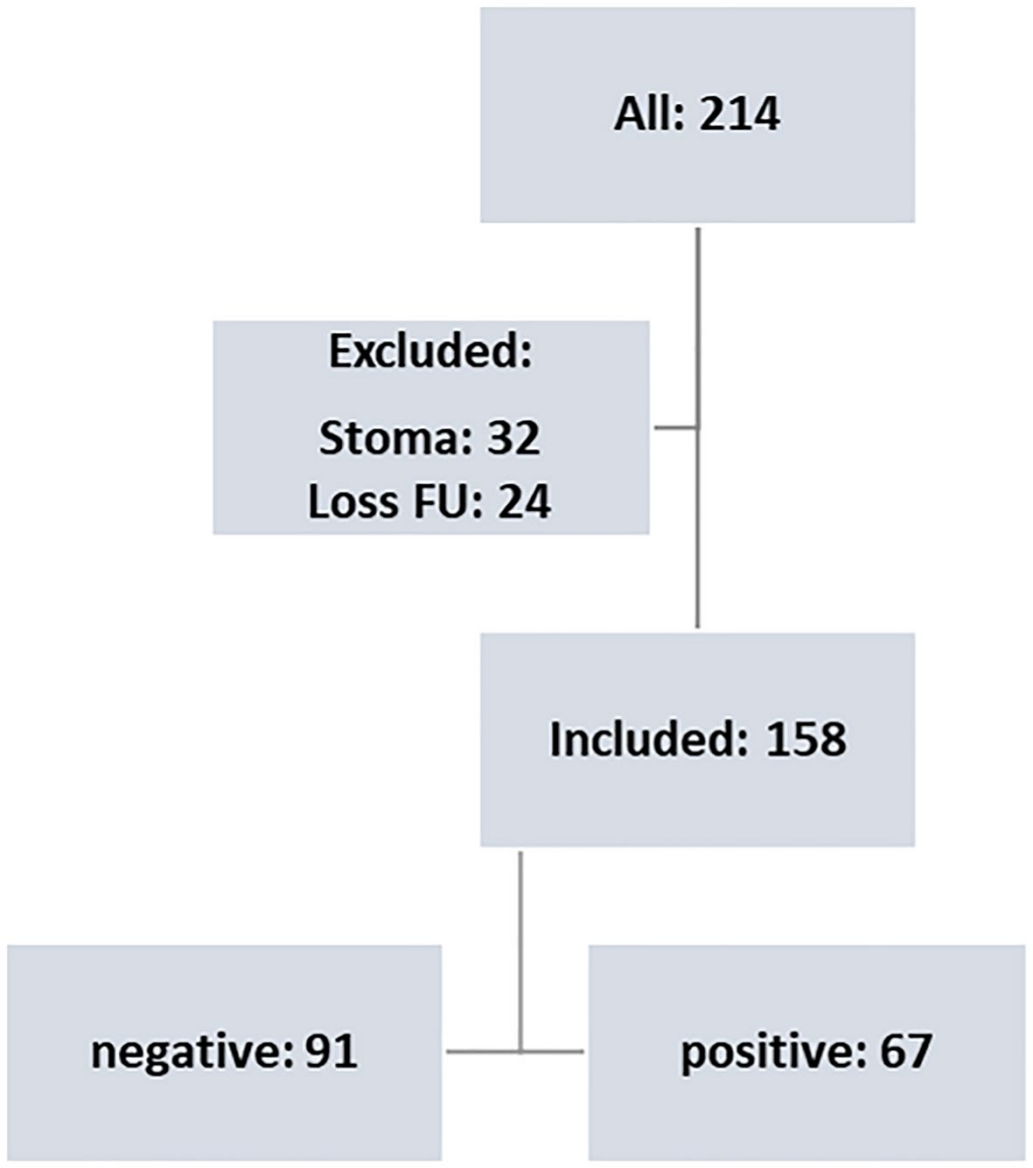
Study design.

From 2014 to 2021, 214 patients received ICR at the Department of General Surgery at the University Hospital of Wuerzburg (Fig. 2). After excluding 56 patients due to loss of follow-up or long-term stoma, independent pathological evaluation of surgical specimens revealed positive (inflamed) resection margins in 91 patients and negative (non-inflamed) margins in 67 patients (Fig. 2). Sociodemographic characteristics were comparable between both cohorts concerning sex, age, BMI and smoking habits (Table 1). Importantly, no differences were observed regarding the technical aspects of the anastomosis or preoperative immunosuppressive medication between both groups.

**Table 1:** Patient characteristics.

	Neg. (n = 91)	Pos. (n = 67)	p-value
Sex			
Male	53	36	0.57
Female	38	31	
Age, years, median	37.9	35.3	0.28
BMI, median	23.5	23.2	0.62
ASA classification, median	2.01	2.02	0.79
Smoking, n (%)	28 (30.8)	15 (22.4)	0.24
Previous abdominal surgery, n (%)	19 (20.9)	13 (19.4)	0.82
Preoperative immunosuppressive medication, n (%)			
None	33 (36.3)	28 (41.2)	0.58
Steroids	39 (42.9)	19 (28.4)	
TNF	22 (24.2)	16 (23.9)	
Biologicals	4 (4.4)	12 (17.9)	
Surgical approach, n (%)			
Laparoscopic	52 (57.1)	39 (58.2)	0.89
Open	39 (42.9)	28 (41.8)	
Anastomosis, n (%)			
Side-to-side	32 (35.2)	26 (38.8)	0.60
Side-to-end	34 (37.4)	20 (29.8)	
Kono-S	14 (15.4)	12 (17.9)	
End-to-end	2 (2.2)	0	
Stoma	9 (9.9)	9 (13.4)	
Anastomotic leakage, n (%)	6 (5.6)	7 (9.9)	0.39
CCI	6.8	7.2	0.86

### Short-term postoperative outcome

Regarding short-term outcome of perioperative morbidity, rates of anastomotic leakages (5.6% versus 9.9%, p = 0.39) were comparable between both groups. In addition, the number of overall complications as demonstrated by the comprehensive complication index (CCI) did not demonstrate an effect of positive resection margins on short-term postoperative outcome (6.8 versus 7.2, p = 0.86) (Table 1).

### Disease recurrence

For the evaluation of the primary and secondary outcomes, 158 patients were finally included for further analysis of which 91 patients had negative resection margins and in 67 patients microscopic disease was detected. Regarding the primary outcome of severe endoscopic disease recurrence during follow-up, patients with positive resection margins had significantly increased rates of severe endoscopic recurrence (Rutgeerts-score i3-i4) compared to patients with negative margins (4.2% versus 19.6%, p = 0.001). The median follow-up was comparable between both groups (33.0 months versus 34.1 months, p = 0.99) (Table 2). In line with that, positive resection margins were identified as independent risk factor for severe endoscopic disease recurrence in a multivariate analysis (p = 0.015, OR 5.94, 95% CI 1.41–25.13) (Table 4).

**Table 2:** Overall follow up.

	Neg. (n = 91)	Pos. (n = 67)	p-value
Follow up, months	33.0	34.1	0.99
Rutgeerts-score, median	1.23	1.18	0.79
Severe endoscopic recurrence (i3-i4), n (%)	3/71 (4.2)	10/51 (19.6)	**0.001**
Clinical recurrence, n (%)	17 (18.5)	13 (19.4)	0.88
Surgical recurrence, n (%)	0 (0)	3 (4.5)	**0.04**
Immunosuppressive medication/biologicals, n (%)	57 (61.9)	48 (71.6)	0.24

Significant values are given in bold.

Regarding secondary outcomes, no differences were seen for median Rutgeerts-scores for patients with negative and positive resection margins (1.23 versus 1.18, p = 0.73). However, severe endoscopic disease recurrence (Rutgeerts-score i3-i4) was significantly increased after 6 months in patients with positive resection margins compared to patients with negative resection margins (2.0% versus 15.6%, p = 0.02). Overall rates of endoscopic recurrence (i1-i4) were comparable between both groups after 6 months (65.3% versus 65.6%, p = 0.98) and rates of postoperative medical prophylaxis were similar as well (28.5% versus 34.3%, p = 0.59) (Table 3). During overall follow-up, while no differences were identified for rates of clinical recurrence (18.5% versus 19.4%), rates of surgical recurrence were significantly increased in patients with positive resection margins (0% versus 4.5%, p = 0.04). Regarding the need for immunosuppressive medication postoperatively, no differences were seen between both groups (61.9% versus 71.6%, p = 0.24). In a subgroup analysis, no differences were observed for the site of positive margins (ileum versus colon) on the occurrence of postoperative recurrence (data not shown).

**Table 3:** Six months follow up.

	Neg. (n = 49)	Pos. (n = 32)	p-value
Endoscopic recurrence (i1–i4), n (%)	32 (65.3)	21 (65.6)	0.98
Severe endoscopic recurrence (i3-i4), n (%)	1 (2.0)	5 (15.6)	**0.02**
Postoperative prophylaxis, n (%)	14 (28.5)	11 (34.3)	0.59

Significant values are given in bold.

## Discussion

In Crohn’s disease, postoperative disease recurrence at the site of the anastomosis following ileocecal resection remains a major challenge for all disciplines resulting in a significant burden for many patients. Despite relevant advances of surgical techniques, real world data demonstrate high rates of recurrent inflammation at the site of the anastomosis during follow-up^26^. An important aspect to improve postoperative outcome and to decrease rates of disease recurrence is the role of inflamed resection margins. Here we demonstrate that overall rates of endoscopic recurrence are independent of resection margins, but that rates of severe endoscopic recurrence and surgical recurrence are associated with positive resection margins. Based on this, we identified positive resection margins after ileocecal resection in CD as a risk factor for severe disease recurrence.

Despite the surgical resection of specimens in macroscopically non-inflamed areas, 42.4% of patients had positive resection margins which is in line with previous studies underlining the importance of this issue^27^. After analyzing the effect of resection margins in patients with localized terminal ileitis, positive resection margins were not associated with perioperative morbidity. However, positive resection margins resulted in significantly increased rates of severe endoscopic disease recurrence (Rutgeerts-score i3-i4) not only after 6 months (2.0% versus 15.6%, p = 0.02) but also during overall follow-up (4.2% versus 19.6%, p = 0.001) despite similar rates of postoperative prophylaxis. A multivariate analysis confirmed the relevant role of positive margins as independent risk factor for severe endoscopic recurrence (p = 0.015) (Table 4). Importantly, while no patient with inflammation-free margins needed to be re-operated due to disease recurrence at the site of the anastomosis during follow-up, positive resection margins were significantly associated with increased rates of surgical recurrence (0% versus 4.5%, p = 0.04) (Table 2). These results underline the clinical relevance of positive inflamed resection margins.

**Table 4:** Multivariate analysis of severe endoscopic recurrence (i3/i4).

	p-Value	Odds ratio (OR)	95% CI
Smoking	0.06	3.68	0.96–14.1
Previous abdominal surgery	0.17	0.20	0.02–1.96
Immunosuppressive medication	0.69	1.36	0.30–6.24
Positive resection margins	**0.015**	5.94	1.41–25.13

Significant values are given in bold.

In general, it remains a major challenge to identify patients who benefit most from postoperative prophylaxis while avoiding overtreatment. While European guidelines recommend prophylactic treatment in the presence of at least one risk factor (smoking, penetrating disease, previous surgery)^15^, the American Gastroenterology Association (AGA) recommends early postoperative medical prophylaxis in all patients independently of the presence of risk factors^16^. However, in an additional commentary the authors state that it might be reasonable to select endoscopy-guided pharmacological treatment over medical prophylaxis postoperatively in patients with low risk of disease recurrence to avoid potential adverse events^16^. While a clear definition of “low risk” is lacking and the overall evidence is very low, those statements underline the importance to identify additional risk factors to enable selective treatment while decreasing overtreatment and healthcare costs. No recommendation exists in current guidelines on positive resection margins due to heterogenous evidence and lack of established histopathological criteria for margins positive for CD. On the one hand, different studies and reviews did not observe any influence for microscopic disease at the resection margins on disease recurrence^18,28^. Furthermore, a prospective randomized controlled trial demonstrated that clinical and surgical recurrence rates did not increase in case of active inflammation at sites of resection margins^29^. On the other hand, Riault et al. showed that patients with microscopic disease had increased rates of clinical and surgical recurrence without evaluating endoscopic aspects^21^. Similarly, in a smaller study Poredska et al. revealed that inflammation at resection margins is associated with an increased risk of early postoperative disease recurrence after 6 months^20^. In other studies, Wasmann et al. identified a positive distal colonic resection margin as main risk factor for postoperative recurrence^30^ while Hammoudi et al. demonstrated the relevance of ileal lesions^31^. In addition, two recent meta-analyses supported the negative effect of positive resection margins on clinical and surgical disease recurrence while including patients receiving surgical resection at different localizations resulting in heterogenous patient cohorts^17,27^. However, conclusions of the presented studies are limited due to heterogenous inclusion criteria, operating techniques, time points and histopathological definitions. Importantly and with regard to the latter aspect, no common criteria for positive resection margins exist to date. While various studies used their own histopathological standards in the past investigating an extensive amount of nonspecific histopathological criteria, one major issue remains to distinct CD-associated inflammation at the margins from surgery-related inflammation. To overcome these issues, our study evaluated a homogenous patient cohort and defined relevant histopathological criteria based on an international consensus of pathologists specialized in IBD adjusted from previous studies^20–22^. As visualized in Fig. 1, these highly-specific histopathological criteria focus on the most relevant histological aspects of CD-associated inflammation such as neutrophils localized in the epithelium referred to as cryptitis, and/or plexitis to facilitate a clear differentiation from surgery-induced inflammation. Based on those aspects, our results demonstrate the significance of defined microscopic disease at resection margins on severe disease recurrence.

Bowel-sparing surgery is still a primary dogma in CD due to the risk of re-resections which, at some point, might result in a potential short-bowel syndrome. Therefore, intraoperative histopathological evaluation of resection margins to potentially extend the resection until margins are inflammation free as it is performed in oncological surgery can currently not be recommended. However, based on the increasing evidence about the importance of positive resection margins on postoperative disease recurrence as presented in our larger study, microscopic disease should be considered as risk factor for disease recurrence. Therefore, consensus criteria for the histopathological definition of CD-associated inflammation at resection margins is of great relevance and all patients with positive margins should be evaluated for medical prophylaxis postoperatively. Following that, indication of medical treatment should not only be considered on patient characteristics and disease history but also on histopathological aspects. Based on our observation, the evaluation and awareness of resection margins based on our specified definition can help to identify patients benefiting most from postoperative prophylaxis. Therefore, histopathological reports need to review the mentioned criteria in detail and should be considered for clinical decision making in the future. These aspects hold significant clinical importance as there are no established strategies to evaluate and address positive resection margins.

Our study has several limitations including its retrospective character and the monocentric design. Furthermore, we lost numerous patients during follow-up which is can be explained by the German Health Care system where a large private practice sector takes care of postoperative follow-up visits and treatment strategies. Nevertheless, this study represents one of the largest studies on this issue and constitutes of a homogenous cohort of patients suffering from localized terminal ileitis representing daily clinical routine. In addition, we implement a clear definition of resection margins positive for CD-associated inflammation.

## Conclusion

Postoperative disease recurrence is a relevant clinical issue and the exact pathophysiology of postoperative disease recurrence is poorly understood^3,25^. In addition, general strategies are lacking to address and identify patients at risk. In our study, positive resection margins were significantly associated with increased rates of severe endoscopic recurrence and surgical recurrence. Based on those results, histopathological analyses for resection margins should always be respected and individual patients evaluated for postoperative prophylaxis. We suggest that future guidelines need to list positive resection margins defined by a homogenous definition as risk factor for disease recurrence.

## Data Availability

Data is available on request. Data is provided by the corresponding author.
